# Comparative activities of ampicillin and teicoplanin against *Enterococcus faecalis* isolates

**DOI:** 10.1186/s12866-022-02753-1

**Published:** 2023-01-06

**Authors:** Georgios V. Zacharopoulos, Georgios A. Manios, Marios Papadakis, Dimitra Koumaki, Sofia Maraki, Dimitrios Kassotakis, Eelco De Bree, Andreas Manios

**Affiliations:** 1 Department of Surgical Oncology, University Hospital of Heraklion, Heraklion, Crete, Greece; 2 Department of Computer Science and Biomedical Informatics, University of Thessaly, Lamia, Greece; 3 Department of Surgery II, Witten/Herdecke University, Heusnerstrasse 40, Postal code, 42283 Witten, Germany; 4 Department of Dermatology and Venereology, University Hospital of Heraklion, Heraklion, 71110 Crete, Greece; 5 Department of Clinical Microbiology, University Hospital of Heraklion, Heraklion, Crete, Greece

**Keywords:** *Enterococcus faecalis*, Ampicillin, Teicoplanin, Antibiotic resistance

## Abstract

**Background:**

*Enterococcus faecalis* remains one of the most common pathogens causing infection in surgical patients. Our goal was to evaluate the antibiotic resistance of *E. faecalis*, causing infections in a surgical clinic, against two antibacterial drugs, ampicillin and teicoplanin. One commonly administered in the past for such infections, ampicillin, and another newer, teicoplanin, which demonstrated exceptionally good efficacy.

**Methods:**

Data from 1882 isolates were retrieved from the microbiology department database during two 5-year periods. Standard biochemical methods were employed for the identification of the isolates. The prevalence of *E. faecalis* among patients with clinical evidence of infection in a surgical oncology ward was assessed. Confidence interval (CI) as well as standard error (SE) were calculated. Moreover, the annual incidence of *E. faecalis* infections in this surgical ward was recorded. The susceptibility of *E. faecalis* to ampicillin and teicoplanin was studied and compared using Fisher’s exact test.

**Results and conclusion:**

Results showed that the incidence of *E. faecalis* infections in the surgical clinic was increasing. Ampicillin, in the later year period, was not statistically different from teicoplanin in treating *E. faecalis* infections. Consequently, ampicillin seems currently to be an effective antibiotic against such infections that could be used as empiric therapy.

## Introduction

Surgical infection represents one of the most serious complications that patients face during their healing process. It is associated with a higher death rate, longer hospitalization, and more intense post-discharge care [1]. Enterococci are one of the most common bacteria isolated from infections in surgical patients. Phylogenetically, the genus *Enterococcus* belongs to the branch of Gram-positive bacteria. The genus *Enterococcus* consists of several species that occur in human and animal gastrointestinal (GI) tracts, as well as in the guts of insects, traditional fermented food, and dairy products, and in various environments including plants, soil, and water [1–5]. *Enterococcus* is also a nosocomial pathogen with opportunistic behavior. It is responsible for a variety of infections such as wounds, intra-abdominal, urinary tract, catheter-associated infections, suppurative thrombophlebitis, endocarditis as well as pneumonia [6]. During the past few decades, enterococci have emerged as important healthcare-associated pathogens [7–12]. Moreover, antibiotics-resistant *Enterococcus* that are isolated from nosocomial infections is difficult to treat, making the bacterium a challenging issue for clinicians in the twenty-first century [12–14].

As there is a paucity of data regarding the incidence of *E. faecalis* infections and their antimicrobial resistance to ampicillin and teicoplanin in tertiary hospitals in South Greece, the aim of this study was to describe the epidemiological features of *E. faecalis* infections in surgical patients at Heraklion University Hospital in Heraklion, Crete, by outlining their antimicrobial resistance against two commonly used drugs, ampicillin, and teicoplanin. All methods were carried out in accordance with relevant guidelines and regulations.

## Materials and methods

A total of 1882 isolates from wound, blood, and urine cultures were collected over two 5-year periods from patients hospitalized in the department of surgical oncology at the university hospital of Heraklion, Crete. Five hundred eighty-six isolates were collected from 2010 to 2014 and 1296 isolates from 2017 to 2021. All data were retrieved from the Microbiology department database.

Isolates were identified at the genus level by standard biochemical assays (esculin hydrolysis, growth in 6.5% salt broth), and at the species level by the Vitek 2 system (Vitek 2, GP panel; BioMérieux, Marcy l’ Etoile, France), and was confirmed by the matrix-assisted laser desorption time of flight mass spectrometry (MALDI-TOF MS) (version 3.2) (BioMérieux). Susceptibility to antimicrobials was determined using the automated system Vitek2 (BioMérieux) [15, 16]. Results were interpreted according to the Clinical and Laboratory Standards Institute criteria (CLSI) [17].

**Fig. 1:**
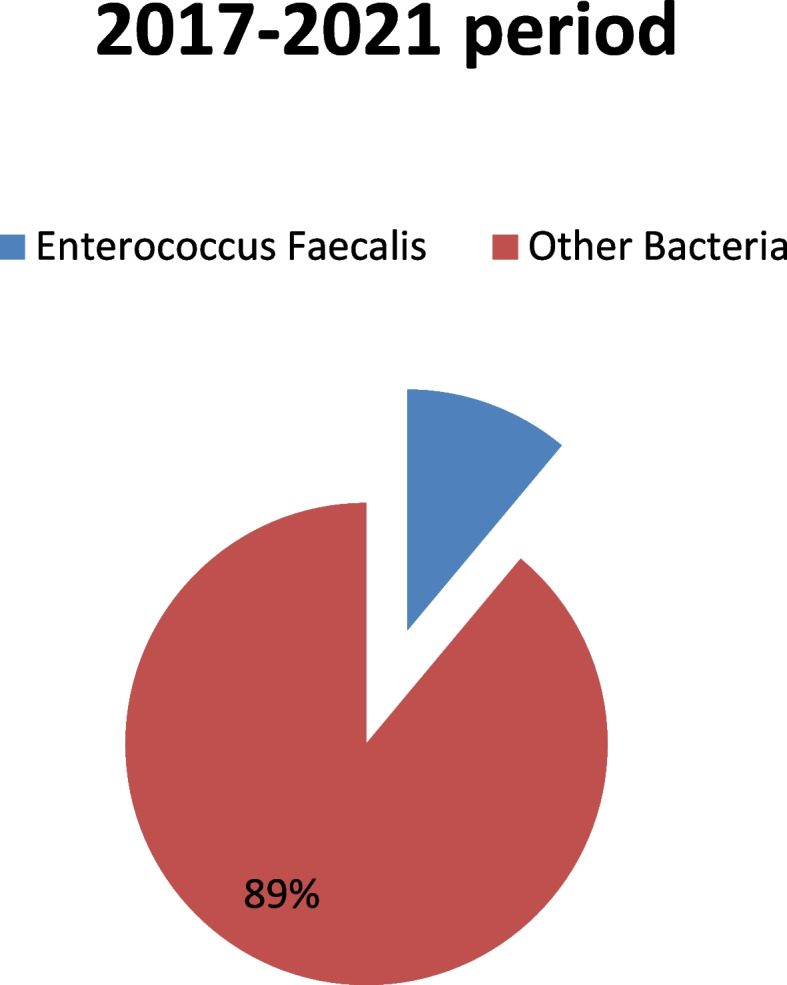
Bacterial ratio of surgical infections in the 2017–2021 period

**Fig. 2:**
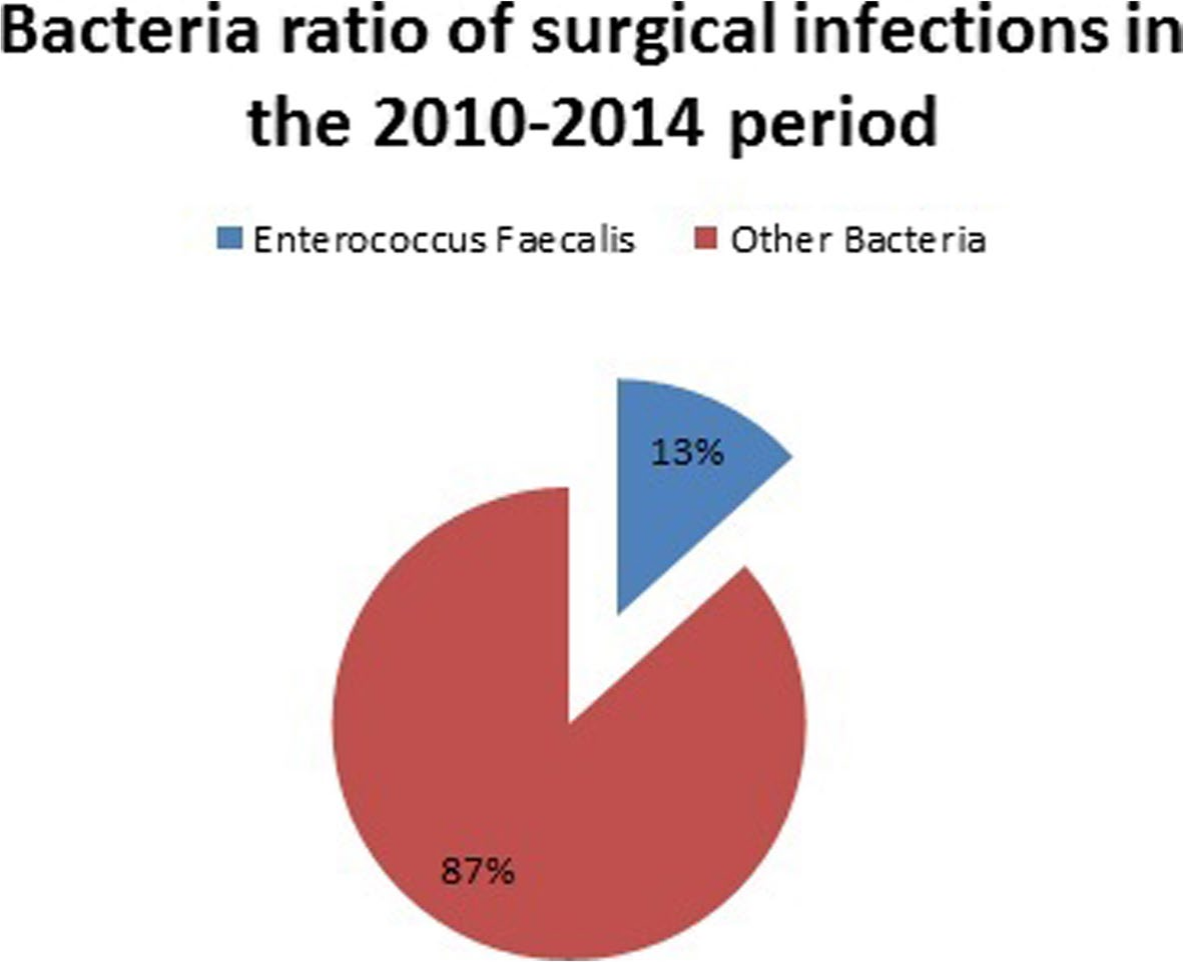
Bacterial ratio of surgical infections in the 2010–2014 period

**Fig. 3:**
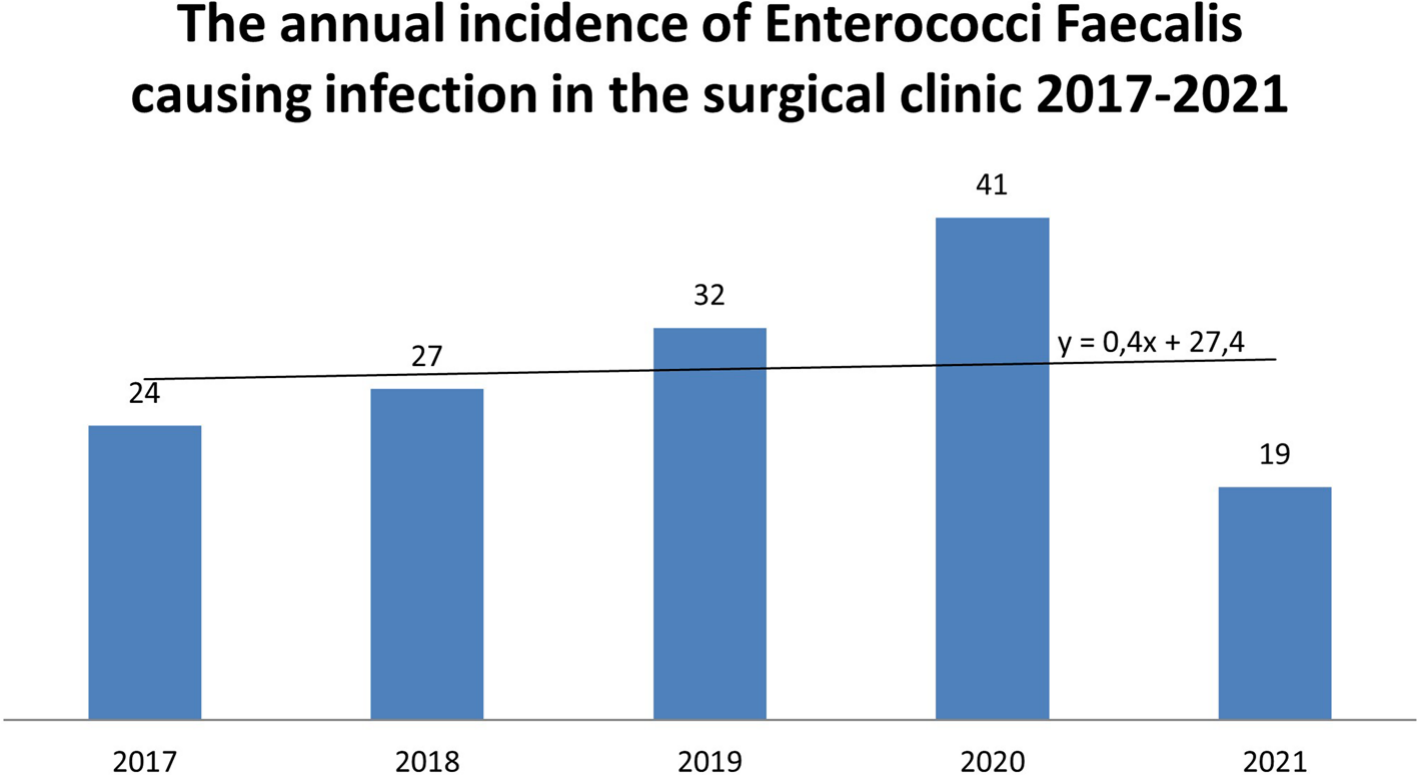
The annual incidence of *Enterococcus faecalis* infections in the surgical clinic 2017–2021 represented also in a line bar diagram

**Fig. 4:**
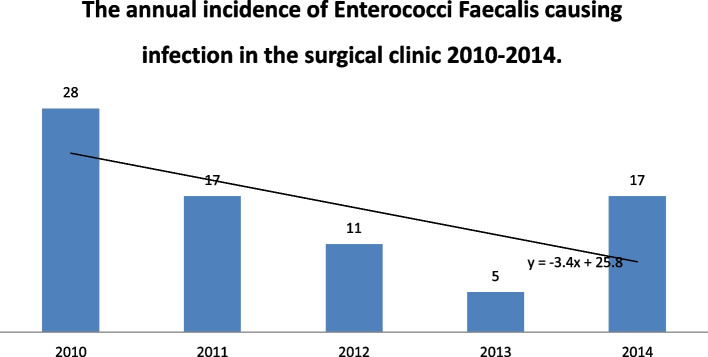
The annual incidence of *Enterococcus faecalis* causing infections in the surgical clinic 2010–2014 represented in a line bar diagram

**Fig. 5:**
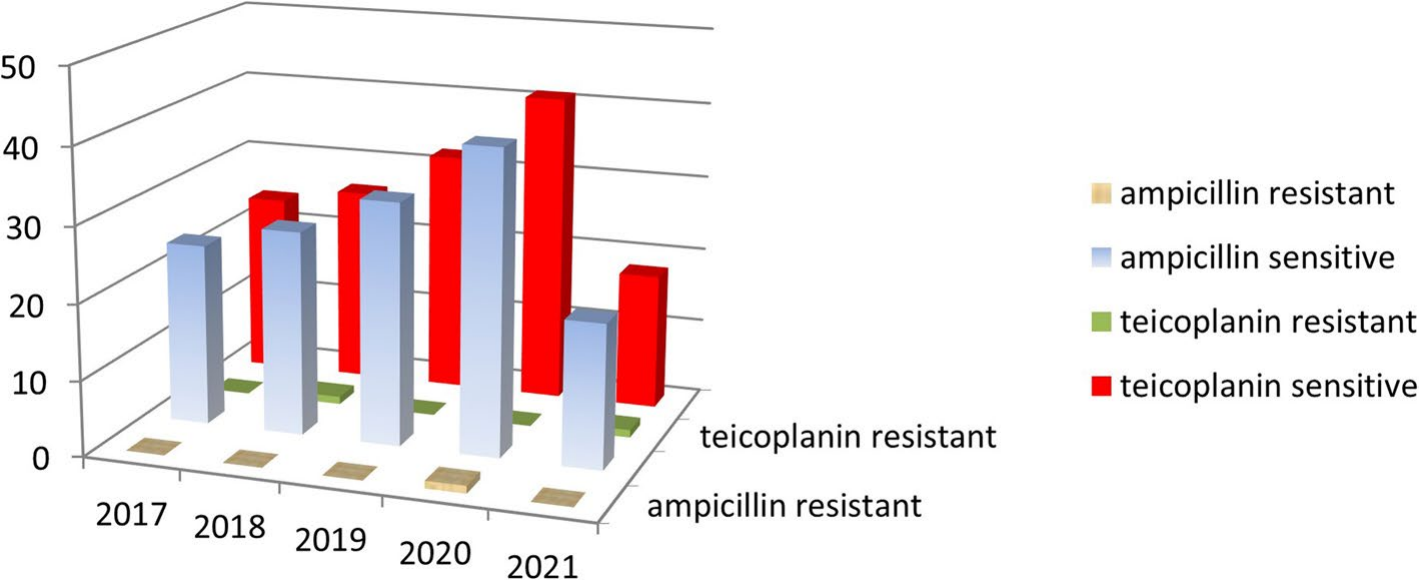
Annual resistance of *Enterococcus faecalis* isolates against ampicillin and teicoplanin 2017–2021

**Fig. 6:**
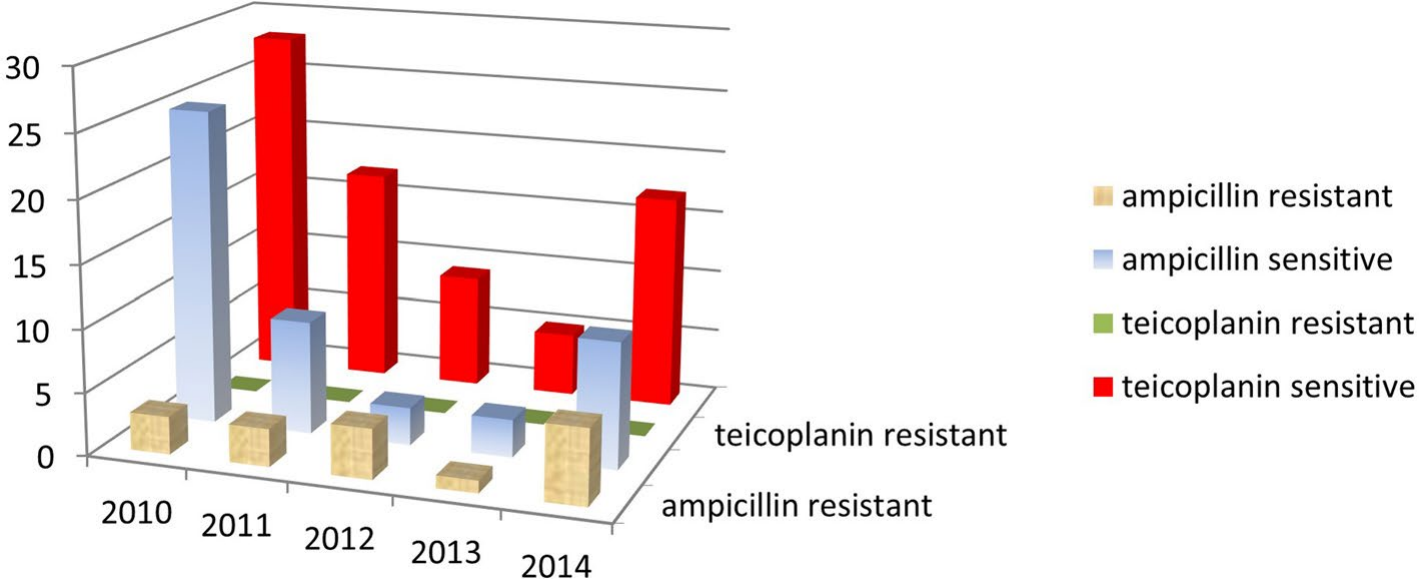
Annual resistance of *Enterococcus faecalis* isolates against ampicillin and teicoplanin 2010–2014

During the 2010–2014 period, the susceptibility to ampicillin was tested in 67 out of 78 Enterococcus isolates while the susceptibility to teicoplanin was in 76 out of 78. In each 5-year period, the percentage of *E. faecalis* infections out of total infections (Figs. 1 and 2), the confidence interval (CI) as well as standard error (SE) were calculated. Moreover, the number of infections with *E. faecalis* per year was recorded. (Figs. 3 and 4) The susceptibility of *E. faecalis* to ampicillin and teicoplanin per year in both periods was calculated (Figs. 5 and 6). Fisher’s exact test was used to compare this susceptibility in these two periods. (Table 1).

**Table 1:** Comparison of the effectiveness of Ampicillin-Teicoplanin against *Enterococcus faecalis* between 2010 and 2014 and 2017–2021

	**Ampicillin** **in 2010–2014**	**Teicoplanin** **in 2010–2014**
Resistant	17 (21.8%)	0
Susceptible	50 (78,2%)	76(100%)
Fisher’s Exact test	*P* < 0.000001	
	**Ampicillin** **in 2017–2021**	**Teicoplanin** **in 2017–2021**
Resistant	1 (0.7%)	2 (1.4%)
Susceptible	142 (99.3%)	141 (98.6%)
Fisher’s Exact test	*P* = 0.5	

## Results

A higher number of enterococcal infections was recorded in the period 2017–2021(143) (Fig. 5) compared to 2010–2014 (78) (Fig. 6). Nevertheless, the percentage of enterococcal infections among all infections remained roughly the same between 2017 and 2021 11.03% [95% CI = 0.1103 + − 1.96*0.0087 = (0.0932,0.1274)] and 2010–2014 (13.31%) [95%CI = 0.1331 + − 1.96*0.0140 = (0.1056,0.1606)] (Figs. 1 and 2).

A decrease in the percentage of resistant *Enterococcus* strains to ampicillin was observed from 21.8% (17 out of 67) in the period 2010–2014 to 0.7% (1 out of 143) in the period 2017–2021. (Table 1).

In the period 2010–2014 teicoplanin was effective against all strains of *E. faecalis* but ampicillin was not (Fig. 6), making the former preferable to administer against *E. faecalis*. (*P* < 0.000001) (Table 1). After 3 years, during the 2017–2021 period, ampicillin was not statistically different from teicoplanin in treating *E. faecalis* infections. (*P* = 0.5) (Table 1).

## Discussion

In our study, a clear increase in *E. faecalis* infections were reported between the two 5-year periods. However, the overall prevalence of *E. faecalis* remained almost stable ranging from 13.31% in the 2010–2014 to 11.03% during the 2017–2021 period. These data were higher than that of the 4-year summary from 2011 to 2014 reported to the national healthcare safety network at the Centers for Disease Control and Prevention of the USA, which was 7.4% [18]. This could be explained by the study participants as the American study included patients from all the departments, various hospitals as well as rehabilitation facilities. The methods employed for the detection of *E. faecalis* could also attribute to the different results. In another study, *E. faecalis* was isolated in 24 of 200 (12%) surgical wound samples and in 2 of 100 (2%) blood cultures [19]. All isolates were resistant to ampicillin and 19.2% were resistant to teicoplanin [19]. The results of our study are in accordance with a recent publication from China that reported that resistance rates to ampicillin decreased gradually but to teicoplanin increased for *E. faecalis* [20].

Ampicillin is a penicillin beta-lactam antibiotic, with effectiveness against most infectious organisms like *E. coli*, *S. pneumoniae*, and *H. influenzae* [21, 22]. Teicoplanin is a glycopeptide antibiotic that was isolated more than 40 years ago as a product of Actionplanes teicomyceticus [23]. It has potent bactericidal activity against a wide variety of aerobic and anaerobic gram-positive bacteria. Its adverse effects include ototoxicity, nephrotoxicity, skin rash, eosinophilia, neutropenia, and transient elevation of serum aminotransferases [24]. It has been shown that serum levels of teicoplanin may not be predictable when administered to seriously ill patients, making cautious use in such cases mandatory [25].

Presently, the treatment of enterococcal infections represents one of the most arduous problems that physicians are dealing with. An increased prevalence of strains that are resistant to almost all antibiotics with in vitro bactericidal activity against enterococci has been observed, reflecting a perturbing tendency. The enterococci are intrinsically resistant to many commonly used antimicrobial agents, namely cephalosporins, aminoglycosides, clindamycin, quinupristin/dalfopristin, and trimethoprim-sulfamethoxazole [26]. All enterococci exhibit decreased susceptibility to penicillin and ampicillin, as well as high-level resistance to most all semi-synthetic penicillins [27]. Nonetheless, for many isolates, despite their level of resistance to ampicillin, its clinical use is not prohibited. Actually, ampicillin remains the treatment of choice for enterococcal infections that lack other mechanisms for high-level resistance [27]. Our data are in concordance with this therapeutic trend indicating that, currently, ampicillin could be effective against *E. faecalis* isolates. In the second 5-year period both ampicillin and teicoplanin remained highly active against *E. faecalis* isolates.

Vancomycin-resistant enterococci (VRE) are a usual and difficult-to-treat reason for hospital-acquired infection [28]. VRE are distinguished from other strains of *Enterococcus* by a raised minimum inhibitory concentration (MIC) for vancomycin and the presence of vancomycin-resistance gene clusters such as *vanA* [29]. High-level resistance to vancomycin is encoded by different clusters of genes referred to as the vancomycin-resistance gene clusters (for example, *vanA*, *B*, *D*, and *M* gene clusters). VanA is the most common type of vancomycin resistance, usually mediates higher levels of resistance than other types, and causes cross-resistance to teicoplanin. The VanB phenotype, the second most common type, is less frequently encountered than VanA [30].

High-level vancomycin resistance is the most problematic resistance of enterococci because it often appears in strains already highly resistant to ampicillin. The three major phenotypes, VanA, VanB, and VanD, can sometimes be differentiated by the level of vancomycin resistance, susceptibility to the glycopeptide antibiotic teicoplanin, and whether the resistance is induced by exposure to teicoplanin [30].

Adherence to protocols for cleaning patient rooms should be monitored to decrease environmental contamination with VRE [31]. Healthcare-associated VRE is transmitted in the hands of healthcare workers; as a result, good hand hygiene is considered an essential measure for reducing the spread of this pathogen. Colonization with VRE typically precedes infection. Colonization most commonly occurs in patients with previous antimicrobial therapy and residents in long-term care facilities [32].

A multimodal strategy is required for VRE prevention and control, including general infection prevention measures including the best care of vascular and urinary catheters, accurate and quick diagnosis and management, judicious use of antibiotics, and infection transmission prevention [32–34].

.An infection control program is essential to surgical site infection (SSI) prevention [35]. A successful program may decrease the rate of SSIs by 40% [27–30]. The prompt administration of efficient preoperative antibiotics and careful attention to surgical technique rank as the most crucial elements in the prevention of SSI, along with maintaining a clean operating room environment. A variety of topical and local antibiotic delivery methods as well as wound-protecting barrier devices have been employed during surgery to lower the incidence of SSI [36]. .The use of antimicrobial-coated sutures may minimize the risk of SSI, although the available and high-quality data are scarce [37].

## Conclusions

Our study suggests that ampicillin could reprise its role as a first-line treatment of enterococcal infections with teicoplanin reserved for cases in which the former cannot be used, such as due to a β-lactam allergy. Clearly, larger and more meticulous studies are necessary to confirm the role of ampicillin. What is apparent from our study is that the resistance of *E. faecalis* to ampicillin does not remain stable but weakens over a relatively short period of time. Thus an antibiotic that in one period may not show increased activity against this pathogen in another period of time may still be suitable and effective for the treatment of *E. faecalis* infections. Ampicillin may be a suitable agent for treating infections caused by *E. faecalis*. Further research is necessary to validate these results and establish its use as empiric therapy against such infections.

## Data Availability

The data that supports the findings of this study are available upon reasonable request from the corresponding author.

## Abbreviations

*E. faecalis*: *Enterococcus faecalis*
CI: Confidence interval
SE: Standard error
VRE: Vancomycin-resistant enterococci
MIC: minimum inhibitory concentration
SSI: surgical site infection
